# Fine-scale spatial and temporal variations in insecticide resistance in *Culex pipiens* complex mosquitoes in rural south-eastern Tanzania

**DOI:** 10.1186/s13071-019-3676-4

**Published:** 2019-08-23

**Authors:** Nancy S. Matowo, Said Abbasi, Givemore Munhenga, Marcel Tanner, Salum A. Mapua, David Oullo, Lizette L. Koekemoer, Emanuel Kaindoa, Halfan S. Ngowo, Maureen Coetzee, Jürg Utzinger, Fredros O. Okumu

**Affiliations:** 1 Environmental Health and Ecological Sciences Department, Ifakara Health Institute, Ifakara, Tanzania; 2 Swiss Tropical and Public Health Institute, Basel, Switzerland; 3 University of Basel, Basel, Switzerland; 4 Department of Disease Control, London School of Hygiene and Tropical Medicine, London, UK; 5 Wits Research Institute for Malaria, Wits/SAMRC Collaborating Centre for Multi-Disciplinary Research on Malaria, School of Pathology, Faculty of Health Sciences, University of the Witwatersrand, Johannesburg, South Africa; 6 Centre for Emerging Zoonotic & Parasitic Diseases, National Institute for Communicable Diseases, Johannesburg, South Africa; 7 US Army Medical Research Directorate-Africa, Kisumu, Kenya; 8 Institute of Biodiversity, Animal Health and Comparative Medicine, University of Glasgow, Glasgow, UK; 9 School of Public Health, Faculty of Health Sciences, University of the Witwatersrand, Johannesburg, South Africa

**Keywords:** *Culex pipiens* complex, Fine spatial scale and temporal differences, Insecticide resistance, Metabolic resistance, Tanzania

## Abstract

**Background:**

*Culex* mosquitoes cause considerable biting nuisance and sporadic transmission of arboviral and filarial diseases.

**Methods:**

Using standard World Health Organization procedures, insecticide resistance profiles and underlying mechanisms were investigated during dry and wet seasons of 2015 and 2016 in *Culex pipiens* complex from three neighbouring administrative wards in Ulanga District, Tanzania. Synergist tests with piperonyl butoxide, diethyl maleate, and triphenyl phosphate, were employed to investigate mechanisms of the observed resistance phenotypes. Proportional biting densities of *Culex* species, relative to other taxa, were determined from indoor surveillance data collected in 2012, 2013, and 2015.

**Results:**

Insecticide resistance varied significantly between wards and seasons. For example, female mosquitoes in one ward were susceptible to bendiocarb and fenitrothion in the wet season, but resistant during the dry season, while in neighbouring ward, the mosquitoes were fully susceptible to these pesticides in both seasons. Similar variations occurred against bendiocarb, DDT, deltamethrin, and lambda-cyhalothrin. Surprisingly, with the exception of one ward in the wet season, the *Culex* populations were susceptible to permethrin, commonly used on bednets in the area. No insecticide resistance was observed against the organophosphates, pirimiphos-methyl and malathion, except for one incident of reduced susceptibility in the dry season. Synergist assays revealed possible involvement of monooxygenases, esterases, and glutathione S-transferase in pyrethroid and DDT resistance. Morphology-based identification and molecular assays of adult *Culex* revealed that 94% were *Cx. pipiens* complex, of which 81% were *Cx. quinquefasciatus*, 2% *Cx. pipiens*, and 3% hybrids. About 14% of the specimens were non-amplified during molecular identifications. Female adults collected indoors were 100% *Cx. pipiens* complex, and constituted 79% of the overall biting risk.

**Conclusions:**

The *Cx. pipiens* complex constituted the greatest biting nuisance inside people’s houses, and showed resistance to most public health insecticides possible. Resistance varied at a fine geographical scale, between adjacent wards, and seasons, which warrants some modifications to current insecticide resistance monitoring strategies. Resistance phenotypes are partly mediated by metabolic mechanisms, but require further evaluation through biochemical and molecular techniques. The high densities and resistance in *Culex* could negatively influence the acceptability of other interventions such as those used against malaria mosquitoes.

## Background

Culicine mosquitoes, including *Aedes*, *Mansonia*, and members of the *Culex pipiens* and *Cx. univittatus* complexes, are common across East Africa [1–4]. Of particular importance is the *Cx. pipiens* complex, generally referred to as the “house mosquito” [5]. It is not only a major cause of biting annoyance to humans but is also a primary vector of many arboviruses and filarial worms that affect more than 1 billion people globally [6–8]. The diseases of concern include Rift Valley fever, dengue, chikungunya, yellow fever, Sindbis, Wesselsbron, o’nyong-nyong, and West Nile arboviruses, filarial worms causing Bancroftian filariasis [6–8], and avian *Plasmodium* species [9]. Most of these pathogens are maintained in zoonotic cycles with humans being incidental hosts [10]. Culicines are adapted and dominate human habitats, increasing their risks to act as bridge vectors in transmitting pathogens between humans and animals [9, 11, 12]. In Africa there have been several sporadic outbreaks of arbovirus infections such as Rift Valley fever in Kenya and Tanzania as well as dengue fever [13–15] and chikungunya [16, 17].

The World Health Organization (WHO) Global Vector Control Response strategy recognizes the need to integrate surveillance and control of pathogens transmitted by different vector species [18]. Surveillance and management of insecticide resistance are two crucial components [18, 19] for effective decision-making on selection, allocation, and implementation of appropriate integrated vector control interventions.

Current vector control interventions in Africa are primarily designed to target malaria vectors, with limited efforts to control other mosquito-borne disease vectors. This is also true for insecticide resistance monitoring [20]. The current emphasis on malaria vectors has resulted in knowledge gaps on species other than *Anopheles* and their resistance profiles to common insecticides used in public health [21, 22]. Yet these species contribute to the greatest human-biting densities. Culicine densities are usually high, because of the presence of numerous favourable aquatic breeding sites that include man-made stagnant water bodies (e.g. small multipurpose dams, rice paddies, etc.), waste disposal sites, open pit latrines and septic tanks, and flooded vegetation [3, 23, 24]. Lack of resources in many countries has limited expansion of surveillance to non-malaria vectors including the culicines.

Previous studies showed spatial and temporal dynamics of insecticide resistance in mosquito vector populations, and influence of environmental contamiants such as agricultural pesticide residues, and such information has been used to plan resistance monitoring efforts [25–27]. In Tanzania, however, insecticide resistance monitoring is carried out at district level in selected sentinel sites in regions assumed to represent different eco-epidemiological settings [28]. Reports from these assessments provide essential data for country-level decision making. However, such a simplistic approach is inadequate for understanding insecticide resistance, which often varies geographically at finer scales other than at the unit of the district or country level [29].

Besides, data on insecticides resistance and associated mechanisms in *Culex* species are also lacking in Tanzania. Synergist assays have been deployed as a quick and simple method to assess metabolic resistance in mosquito vectors [30–33]. Synergists act by enhancing insecticides penetration into the mosquito body and inhibit the metabolic enzymes that would otherwise digest the insecticides, hence partially/fully restoring susceptibility [30–33].

In addition, data on insecticide resistance in male mosquito populations are limited inspite of both males and females being exposed to insecticides during vector control interventions. Male mosquito populations substantially contribute in the reproduction and increasing population density and their response to insecticides is also a crucial component. In addition, novel vector control interventions such as spraying of swarms [34] with insecticides directly target male mosquitoes. This suggests the need to monitor insecticide resistance on a regular basis in male mosquitoes.

We investigated the spatial and seasonal variations in susceptibility to insecticides of *Cx. pipiens* complex mosquitoes from rural south-eastern Tanzanian villages where there is a high coverage of long-lasting insecticidal nets (LLINs) [35], and a regular usage of agricultural pesticides (Matowo et al., unpublished data). The main objectives of the study were (i) to fill important knowledge gaps on insecticide resistance and species diversity of *Culex* mosquitoes in the study area; and (ii) to investigate fine-scale spatial and temporal differences in resistance and resistance mechanism in the *Culex* species.

## Methods

### Study area

**Fig. 1:**
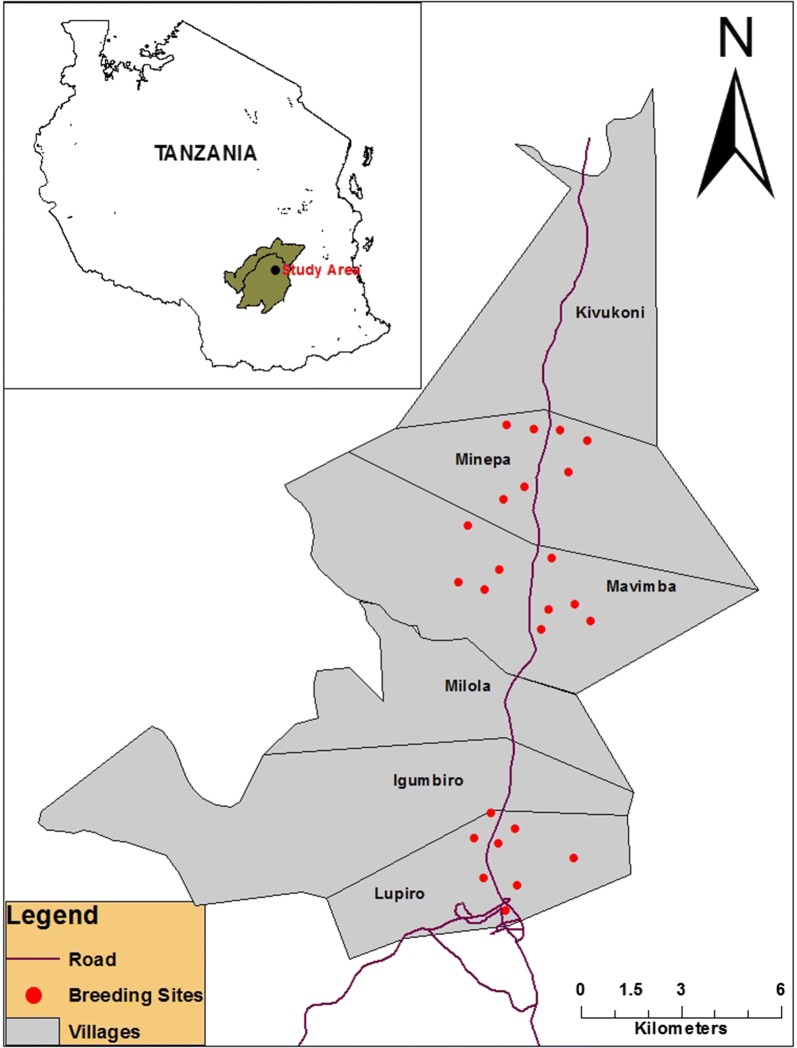
Locations of mosquito aquatic breeding sites in Minepa, Mavimba, and Lupiro, south-eastern Tanzania, where larvae sampling was conducted between June 2015 and June 2016

Three neighbouring wards, i.e. Minepa (8.271°S, 36.677°E), Lupiro (8.385°S, 36.670°E), and Mavimba (8.312°S, 36.677°E), in Ulanga District, south-eastern Tanzania were selected (Fig. 1). These villages have high coverage of LLINs [35] and high agricultural pesticide use for crop protection (Matowo et al., unpublished data). Minimum and maximum distances between the wards was ~ 4 km (Minepa to Mavimba) and ~ 9 km (Minepa to Lupiro). All three wards lie at an altitude between 120 m and 350 m above mean sea level. Average annual precipitation ranges between 1200 mm and 1800 mm, with the dry season between June and October, a short rainy season in November and December, and the wet season between January and May. Mean daily temperatures over the year vary from 20 °C to 32 °C, while the relative humidity is 70–90%. Residents practice rice farming [36], which is irrigated during the dry season, so that the area is continuously favourable for mosquito breeding [37]. A national insecticide susceptibility survey in 2011 across 14 districts, including the nearby Kilombero District, reported widespread pyrethroid and DDT resistance in *Anopheles* mosquitoes [28], but no data on *Culex* were reported. Recent studies indicated that the two malaria vectors *An. arabiensis* and *An. funestus* are highly resistant to pyrethroids, bendiocarb, and DDT, thus compromising vector control efforts [29, 38, 39].

### Mosquito sampling and larval rearing

Mosquito larvae were collected between June 2015 and June 2016, using a standard larval dipping method [40] in three wards, during the dry season (June to December 2015) and wet season (January to May 2016). In each instance, seven to nine randomly selected and georeferenced aquatic habitats were sampled. Larvae were separated into anophelines and culicines to ensure easy adult morphological identification. To assess spatial variations in insecticide resistance, collected larvae were separated per collection site for WHO insecticide resistance assays.

Collected larvae were transferred to the medical entomology laboratory, the “Vector Sphere”, at Ifakara Health Institute (IHI; Ifakara, Tanzania), and reared to adults at temperatures of 27 ± 2 °C and relative humidity of 70–90%). Larvae were fed on mud and algae from their original habitats, supplemented with Tetramin fish food (Tetra; Melle, Germany). Emergent adults were separated by sex and taxa, and provided with 10% glucose solution.

### Insecticide susceptibility tests

Phenotypic insecticide resistance in *Culex* species in the three study villages was assessed in the dry and wet seasons using standard WHO test kits (Vector Control Research Unit, School of Biological Sciences, Universiti Sains Malaysia, Penang, Malaysia). Adult males and females (3–5 days-old) were exposed in batches of either 20 or 25 individuals according to the discriminating doses of 0.05% lambda-cyhalothrin, 0.05% deltamethrin, 0.75% permethrin, 4% DDT, 4% dieldrin, 0.1% bendiocarb, 0.1% propoxur, 0.25% pirimiphos-methyl, and 5% malathion [41]. The same number of mosquitoes were exposed to oil-impregnated papers as controls. Due to unavailability of reference susceptible *Culex* mosquitoes, a susceptible colony of *An. gambiae* (*s.s.*) (Ifakara strain), was used to validate efficacy of test papers. Knockdown was recorded after 10, 15, 20, 30, 40, 50 and 60 min. After the 60 min exposure, mosquitoes were transferred to holding tubes and offered 10% glucose. Final mortality was recorded 24 hours post-exposure [41], after which the mosquitoes were preserved in 1.5 ml Eppendorf tubes containing silica gel for further species identification, using polymerase chain reaction (PCR) assays.

### Synergist assays

Synergist assays were performed using 4% piperonyl butoxide (PBO), a known inhibitor of monooxygenase, 20% diethyl maleate (DEM), an inhibitor of glutathione S-transferases (GSTs), and 20% triphenyl phosphate (TPP), an inhibitor of esterases, as a quick and simple method to assess whether the observed phenotypic resistance had a metabolic enzymes basis [30]. The bio-efficacy of synergist papers was tested against a reference laboratory colony (*An. funestus*) with resistance phenotype mediated by monooxygenases and GSTs [42]. Due to resource limitations, the synergist tests were performed only on female mosquitoes in the dry season in Minepa and Mavimba wards. For each synergist, five cohorts of adults (*n *= 125) were used. The first group was exposed to a synergist (either 4% PBO, 20% DEM, or 20% TPP) for 60 min, and thereafter immediately exposed to WHO test papers impregnated with either 0.75% permethrin, 0.05% deltamethrin, 0.05% lambda-cyhalothrin, or 4% DDT for another 60 min. The second group was exposed only to the respective WHO test papers, and the third group exposed to the synergist only. Fourth and fifth groups consisted of controls, i.e. filter papers treated with olive oil used to prepare the synergist papers (solvent control), and plain filter papers (environmental control).

### Estimating relative densities of *Culex* mosquitoes and associated biting risk

The relative proportion of population densities of female *Culex* species, relative to other mosquito species, was estimated from indoor night collections in 2012, 2013, and 2015 at Minepa, Mavimba, and Kivukoni wards [43, 44], using CDC light traps [45] in 96 randomly selected houses. The mosquitoes were segregated as *Anopheles*, *Culex*, *Aedes*, *Mansonia*, and other species. The proportion of female *Culex* population density was used as a proxy for estimating human-biting risk.

### Morphological identification of *Culex* species

A sub-sample of female *Culex* mosquitoes (*n* = 430) from the resistance bioassays and female *Culex* mosquitoes from indoor collections (*n* = 1053) were morphologically identified to determine composition of prevailing species and species complexes using the taxonomic keys of Edwards [46], under a stereo-zoom microscope (SZM-LED2, digital Optika^®^ Microscopes; Ponteranica, Italy). To improve identification, the mosquito images were enhanced using OptikalSview software version 3.6.6, and captured using a digital camera (Optika^®^; Ponteranica, Italy) attached to the microscope.

The diagnostic features used for species identification were: (i) presence and number of mesepimeral bristles; (ii) presence or absence of a pale band on proboscis; (iii) presence or absence of white scales on abdomen; (iv) presence or absence of white scales on femur or tibia; and (v) presence or absence of pale-ringed tarsi. To complete the assessment, we first adapted the 1941 morphological keys [46] to focus on just the general diagnostic features and specific features known to occur in Tanzania *Culex* mosquitoes (Table 1).

**Table 1:** Identification keys showing main morphological features to distinguish among female *Culex* collected in three rural wards (Minepa, Mavimba, and Lupiro) in Ulanga District, south-eastern Tanzania. Adapted from the morphological keys by Edwards [46]

Taxon	Main morphological features for identification of *Culex* spp.
*Culex pipiens* complex	Generally smaller size compared to other *Culex* species Abdominal tergite with pale basal bands, sternite pale and not banded Proboscis without a well-defined ring in the middle but pale beneath Legs and tarsi mostly or entirely dark but hind tibia with a small pale spot at tip Presence of one lower mesepimeral bristle Halters yellowish
*Culex* (*Lutzia*) *tigripes*	One of the largest *Culex* species About 10 small prominent pale spots on a dark ground marking on femora and tibiae Abdominal bands, 6 and 7 broad, sometimes occupying almost half of the tergites; all sternites pale-scaled, un-banded Mainly dark proboscis Dark-scaled wings 3–10 bristles on lower half of the mesepimeron in a more or less regular row
*Culex* (C*ulex*) *poicilipes*	Sharply-defined median pale yellowish ring on proboscis Presence of 7–10 distinct small pale spots on anterior surfaces of front femora and tibiae Tarsi with pale rings at joints, which are scarcely longer than wide; on joint 4–5 of hind tarsi, pale ring scarcely noticeable No post-spiracular or pre-alar scales Wings with all dark scales
*Culex* (C*ulex*) *duttoni*	Distinctly pale rings on the tarsi and indefinitely ringed proboscis but with whitish scales on the palp almost half Middle tibia with narrow pale anterior stripe Presence of 2–4 lower mesepimeral bristles Presence of few post-spiracular scales Dark thorax with no pale scales Head with pale scales

### Molecular identification of sibling species in the *Cx.* *pipiens* complex

Morphological identification showed that the *Cx. pipiens* complex was the most common of all *Culex* species in the study area. Further molecular identification was conducted using PCR amplification to differentiate two members of the *Cx. pipiens* complex (i.e. *Cx. pipiens pipiens* and *Cx. quinquefasciatus*). This PCR targets the acetyl-cholinesterase-2 locus (*ace-2*). The *ace-2* locus was amplified using primers B126, *ACEquin*, and *ACEpip* as previously described by Smith & Fonseca [47].

DNA was extracted from 280 specimens, randomly selected from the morphologically identified *Cx. pipiens* complex. A total of 5 µl of extracted genomic DNA per sample was amplified in a 20 µl reaction mix containing 1× PCR buffer, 250 µM dNTP, 2 mM MgCl_2_, 0.4 µM of universal primer and *ACEquin*, 0.2 µM of *ACEpip*, and 1 unit of Taq DNA polymerase overlaid by a drop of mineral oil. After PCR amplification, 10 µl of the DNA fragments were separated by electrophoresis on a 2.5% agarose gel stained with 0.5 µg/ml ethidium bromide and compared against a 100-bp DNA marker included in the gel. Separated DNA fragments were photographed under ultraviolet light using Kodak Gel Logic 100 imaging system and scored as *Cx. pipiens pipiens* (610 bp) or *Cx. quinquefasciatus* (274 bp).

### Statistical analysis

The data on susceptibility to insecticides were interpreted following the WHO thresholds established in 2016 [41], where: (i) mean mortality ranging between 98% and 100% indicates susceptibility; (ii) mean mortality between 90% and 97% indicate possible resistance or presence of resistant genes in the vector populations, but requiring confirmation by repeat bioassays or by a molecular assay; and (iii) mean mortality less than 90%, indicates confirmation of resistance in the test populations. Percentage mean mortality for controls were also calculated, and any tests with mortality greater than 5%, but less than 20%, were corrected using Abbott’s formula [48]. Further analysis was done using R statistical software version 3.0 [49]. Mean mortalities in mosquitoes collected either in the dry or wet season were compared using *t*-test and any differences considered statistically significant at *P* < 0.05. In the synergist tests, the observed 24 hours post-exposure was compared between synergised and un-synergised exposures using *t*-test and any differences considered statistically significant at *P* < 0.05. The proportion of female *Culex* mosquito population density relative to other mosquito species were summarised in a tabular format and used as a proxy for estimating exposure of human to bites.

## Results

### Morphological identification of *Culex* mosquitoes

A sub-sample of 430 specimens reared from larvae from the three wards (Table 2) were identified as belonging to four *Culex* species or species complexes as follows: 94% (*n* = 405) *Cx. pipiens* complex; 2% (*n* = 8) *Cx.* (*Lutzia*) *tigripes*; 1% (*n* = 3) *Cx.* (*Culex*) *poicilipes*; and 3% (*n* = 14) *Cx.* (*Culex*) *duttoni*. The 1053 *Culex* mosquitoes sub-sampled from indoor collections were also identified as members of the *Cx. pipiens* complex. Given the dominance of *Cx. pipiens* complex, results of insecticide resistance tests are considered most representative of this species complex.

**Table 2:** Number of adult *Culex* of different species or species complexes identified from sub-samples emerged from larvae collected in three study wards in Ulanga District, Tanzania, in 2015 and 2016

Species	Study wards			
	Minepa ward	Mavimba ward	Lupiro ward	Total
*Cx. pipiens* complex	160	112	133	405
*Cx.* (*Lutzia*) *tigripes*	4	1	3	8
*Cx.* (C*ulex*) *poicilipes*	1	2	0	3
*Cx.* (C*ulex*) *duttoni*	11	0	3	14
Total	176	115	139	430

### Molecular identifications

About 94% of *Culex* belonged to the *Cx. pipiens* complex, of which 81% were verified by PCR as *Cx. quinquefasciatus*, 2% as *Cx. pipiens pipiens*, and 3% as hybrids of *Cx. pipiens pipiens* and *Cx. quinquefasciatus.* A small proportion of samples (14%) did not amplify.

### Insecticides resistance status of *Culex* mosquitoes in different wards and seasons

Table 3 summarizes results for standard WHO susceptibility tests [32] on adult male and female *Culex* in the three study wards. The reference colony (*An. gambiae* (*s.s.*)) used to test insecticidal activity of the test papers was fully susceptible (100%) to all candidate insecticides. No mortality was observed upon exposure of wild-caught *Culex* to untreated papers. The *Culex* mosquitoes sampled displayed differences in resistance to each insecticide by ward, time of year (dry or wet season), sex (male or female mosquitoes), and insecticides tested.

**Table 3:** Fine-scale spatial and seasonal variations in insecticide susceptibility of *Culex* mosquitoes collected in three neighbouring wards in the Ulanga District, Tanzania, in the dry season (June–December 2015) and wet season (January–May 2016). Adult mosquitoes exposed for each insecticide were either 20 or 25 per replicate. Results expressed as % mean mortality 24 hours post-exposure

	Insecticide	Minepa (8.271°S, 36.677°E)		Mavimba (8.312°S, 36.677°E)		Lupiro (8.385º S, 36.670º E)	
		Dry season	Wet season	Dry season	Wet season	Dry season	Wet season
Female mosquitoes	0.75% permethrin^a,b^	100^SS^	100^SS^	100^SS^	100^SS^	100^SS c^	72.0^RR c^
	0.05% deltamethrin	86.0^RR c^	56.3^RR c^	87.0^RR^	90.0^RS^	8.0^RR c^	87.5^RR c^
	0.05% lambda-cyhalothrin	60.0^RR^	82.5^RR^	76.3^RR c^	91.3^RS c^	80.0^RR c^	87.5^RR c^
	4% dieldrin	94.0^RS^	98.8^SS^	98.8^SS^	100^SS^	100^SS^	100^SS^
	4% DDT	92.0^RS^	95.0^RS^	87.5^RR^	91.3^RS^	78.0^RR^	71.0^RR^
	0.1% propoxur	94.0^RS^	100 ^SS^	91.3^RS c^	100^SS^	100^SS^	98.0^SS^
	0.1% bendiocarb^a,b^	29.0^RR c^	99.0^SS c^	98.0^SS^	100^SS c^	100^SS^	99.0^SS^
	0.25% pirimiphos-methyl	100^SS^	100^SS^	90.0^RS c^	100^SS c^	100^SS^	100^SS^
	5% malathion	100^SS^	100^SS^	100^SS^	97.5^SS^	99.0^SS^	100^SS^
	Control (untreated paper)	4.6	2.4	2.2	1.8	1.2	2.9
Male mosquitoes	0.75% permethrin	100^SS^	100^SS^	98.8^SS^	100^SS^	100^SS^	100^SS^
	0.05% deltamethrin^b^	90.0^RS^	93.0^RS^	97.5^RS^	98.8^SS^	99.0^SS^	95.0^RS^
	0.05% lambda-cyhalothrin^a,b^	88.0^RR c^	99.0^SS c^	100^SS^	98.8^SS^	71.0^RR^	92.0^RS^
	4% dieldrin	100^SS^	100^SS^	100^SS^	100^SS^	100^SS^	100^SS^
	4% DDT^a,b^	97.0^RS^	95.0^RS^	77.0^RR^	98.8^SS^	99.0^SS^	100^SS^
	0.1% propoxur	93.0^RS^	99.0^SS^	97.0^RS^	100^SS^	100^SS^	100^SS^
	0.1% bendiocarb^a,b^	58.0^RR c^	100^SS c^	100^SS^	98.0^SS^	100^SS^	100^SS^
	0.25 % pirimiphos-methyl	100^SS^	98.0^SS^	97.0^RS^	100^SS^	100^SS^	100^SS^
	5% malathion	100^SS^	100^SS^	100^SS^	100^SS^	100^SS^	100^SS^
	Control (untreated paper)	1.1	11.2	4.8	2.5	2.6	3.9

*Notes*: Morphological identification of the *Culex* mosquitoes revealed 94% were *Cx. pipiens* complex. Of these, PCR assays revealed that 81% were *Cx. quinquefasciatus*, 2% were *Cx. pipiens pipiens* and 3% were hybrids of the two species. About 14% of the specimens were non-amplified. These test results can therefore be considered primarily representative of *Cx. pipiens* complex or more specifically for *Cx. quinquefasciatus*

*Abbreviations*: SS, mosquitoes were susceptible to the test insecticide (WHO assays mortality between (98% and 100%); RS, mosquitoes had reduced susceptibility indicating possible resistance and need for further investigation (mortality of 90–97%); RR, mosquitoes were confirmed resistant to the test insecticide (WHO assays mortality below 90%)

^a^Chemicals for which we observed differences in susceptibility of *Culex* mosquitoes between dry and wet seasons, i.e. where mosquitoes were fully susceptible in one season and fully resistant in a different season in same ward

^b^Chemicals for which we observed differences in susceptibility of *Culex* mosquitoes between (nearby) wards, i.e. where mosquitoes were fully susceptible in one ward and fully resistant in another ward during the same season

^c^There was a statistically significant difference in mortality between the dry and wet seasons

Overall, lower mortality was observed in the Minepa ward than the other two wards, and females had lower mortalities than males. In addition, resistance to bendiocarb, deltamethrin, lambda-cyhalothrin, and DDT, was higher in the dry season than in the wet season. There was complete resistance or reduced susceptibility to the pyrethroids, except permethrin, against which the mosquitoes (both males and females) from the Minepa and Mavimba wards were fully susceptible regardless of the season. In the Lupiro ward, however, *Culex* were susceptible to permethrin in the dry season, but resistant to it during the wet season. In the Minepa ward, both male and female *Culex* were resistant to bendiocarb in the dry season, but fully susceptible in the wet season. Those *Culex* collected from the Mavimba and Lupiro wards remained fully susceptible to bendiocarb during both seasons. Similar spatio-temporal variations in resistance profiles were observed for male *Culex* exposed to deltamethrin, lambda-cyhalothrin, and bendiocarb.

### Effects of synergists on pyrethroid and DDT resistance phenotypes

Results of synergist tests on the different resistance phenotypes are detailed in Tables 4 and 5. In the Minepa ward, samples synergized with 4% PBO exhibited mean mortality of 57.5% on exposure to 0.05% lambda-cyhalothrin, compared to 35.0% in un-synergized cohorts. The difference in mean mortality was marginal, when examined using two-sample *t*-test (*t*_(6)_ = 2.50, *P* = 0.047). Conversely, synergizing the same population with 20% TPP did not change the mortality after exposure to lambda-cyhalothrin (*t*_(6)_ = 0.23, *P* = 0.827). Resistant phenotype pre-exposure to 20% TPP followed by exposure to deltamethrin, resulted in 1.6-fold increase in mortality, relative to exposure to deltamethrin alone (81.3 *vs* 51.3%). This difference was statistically significant (*t*_(3)_ = 2.84, *P* = 0.030). Similarly, there was a statistically significant difference in mortalities after exposure to 0.05% deltamethrin with or without pre-exposure to 4% PBO (93.8 *vs* 73.8%; *t*_(4)_ = 2.99, *P* = 0.042). However, there was no difference in mortalities in mosquitoes exposed to 4% DDT (90%) with or without pre-exposure to 20% DEM (95.0 *vs* 90.0%; *t*_(6)_ = − 1.73, *P* = 0.134).

**Table 4:** Mean % mortality recorded 24 hours after exposure to lambda-cyhalothrin and deltamethrin, with and without synergist, TPP (triphenyl phosphate) or PBO (piperonyl butoxide). The mosquitoes tested were 3- to 5-day-old adult *Culex* mosquitoes reared from wild-collected larvae from Minepa and Mavimba wards in Ulanga District, Tanzania, in 2015 and 2016

Treatment	No. of replicates	Sample size	Mean % mortality (95% CI)			
			Mavimba (8.312°S, 36.677°E)		Minepa (8.271°S, 36.677°E)	
			0.05% deltamethrin	0.05% lambda-cyhalothrin	0.05% deltamethrin	0.05% lambda-cyhalothrin
Tests with triphenyl phosphate (TPP)						
Environmental control	4	80	0	1.3 (-2.7–5.2)	0	0
Solvent control	4	80	0	0	0	0
20% TPP only	4	80	0	1.3 (-2.7–5.2)	0	0
20% TPP and test insecticide	4	80	86.0 (77.8–94.2)^a^	83.8 (76.1–91.4)^a^	81.3 (63.6–98.9)^a^	73.8 (48.3–99.2)^a^
Test insecticide only	4	80	75.0 (68.9–81.1)^b^	72.5 (62.2–82.8)^b^	51.3 (23.7–79.8)^b^	71.3 (47.5–95.2)^a^
Tests with piperonyl butoxide (PBO)						
Environmental control	4	80	0	0	0	0
Solvent control	4	80	0	1.3 (-2.7–5.2)	0	0
4% PBO only	4	80	0	1.3 (-2.7–5.2)	0	0
4% PBO and test Insecticide	4	80	60.0 (42.8–77.2)^a^	66.3 (54.3–78.2)^a^	93.8 (86.1–101.4)^a^	57.5 (32.8–82.2)^a^
Test insecticide only	4	80	41.3 (33.6–48.9)^b^	28.8 (15.2–42.3)^b^	73.8 (53.9–93.6)^b^	35.0 (20.5–49.5)^b^

*Notes*: Morphological identification of the *Culex* mosquito populations revealed 94% were *Cx. pipiens* complex. Of these, PCR assays revealed that 81% were *Cx. quinquefasciatus*, 2% were *Cx. pipiens pipiens* and 3% were hybrids of the two species. About 14% of the specimens were non-amplified. These test results can therefore be considered primarily representative of *Cx. pipiens* complex or more specifically for *Cx. quinquefasciatus*. Environmental control refers to a control where mosquitoes are exposed to non-treated papers, and is used to assess any contamination in the test environment or during the procedures

^a,b^The letters a and b signify statistically significant differences between % mortalities obtained in tests with or without the synergists

**Table 5:** Mean % mortality recorded 24 hours after exposure to 4% DDT, with and without the synergist, diethyl maleate (DEM). The mosquitoes tested were 3- to 5-day-old adult *Culex* mosquitoes reared from wild collected larvae from Minepa and Mavimba wards in Ulanga District, Tanzania, in 2015 and 2016

Treatment	No. of replicates	Sample size	Mean % mortality (95% CI)	
			Mavimba (8.312° S, 36.677° E)	Minepa (8.271° S, 36.677° E)
Environmental control	4	80	0	0
Solvent control	4	80	0	0
20% DEM only	4	80	0	0
20% DEM and 4% DDT	4	80	82.5 (67.3–97.7)^a^	90.0 (83.5–96.5)^a^
4% DDT only	4	80	48.8 (38.7–58.8)^b^	95.0 (88.5–101.5)^a^

*Notes*: Morphological identification of the *Culex* mosquitoes revealed 94% were *Cx.pipiens* complex. Of these, PCR assays revealed that 81% were *Cx. quinquefasciatus*, 2% were *Cx. pipiens pipiens* and 3% were hybrids of the two species. About 14% of the specimens were non-amplified. Environmental control refers to a control where mosquitoes are exposed to non-treated papers, and is used to assess any contamination in the test environment or during the procedures

^a,b^The letters a and b signify statistically significant differences between % mortalities obtained in tests with or without the synergists

In the Mavimba ward, we observed a significantly higher mortality when mosquitoes were pre-exposed to 20% TPP, followed by lambda-cyhalothrin, as opposed to exposure to lambda-cyhalothrin alone (83.8 *vs* 72.5%; *t*_(6)_ = 2.80, *P* = 0.030). Similarly, pre-exposure to 4% PBO, followed by lambda-cyhalothrin increased mortality relative to exposure to lambda-cyhalothrin alone (66.3 *vs* 28.8%; *t*_(6)_ = 6.60, *P* < 0.001). There was a marginal increase in mortality when mosquitoes were pre-exposed to 20% TPP, followed by exposure to deltamethrin, compared to exposure to deltamethrin alone (86.0 *vs* 75.0%; *t*_(6)_ = 3.42, *P* = 0.014). Pre-exposure to 4% PBO, followed by deltamethrin also resulted in higher mortality relative to cohorts exposed to deltamethrin only (60.0 *vs* 41.3%; *t*_(6)_ = 3.17; *P* = 0.019). Lastly, pre-exposure to 20% DEM, followed by 4% DDT increased mortality in the synergized cohorts, compared to their un-synergized counterparts (82.5 *vs* 48.8%; *t*_(6)_ = 5.89, *P* = 0.001).

### Estimated biting densities of *Culex,* relative to other mosquito species

Of the 387,318 mosquitoes collected indoors during the sampling period, *Culex* constituted 77% (*n* = 299,841) of the total catches. Of these, 79% were females (*n* = 236,484) and 21% males (*n* = 63,375). In total, 1053 *Culex* mosquitoes were subjected to species-specific identification; of these all were identified as members of the *Cx. pipiens* complex (Table 6).

**Table 6:** Relative abundance and indoor distribution of mosquitoes, across three study wards (including Minepa and Mavimba wards, from where *Culex* larvae were also obtained for the resistance tests). Data obtained from an annual mosquito surveillance programme conducted by the Ifakara Health Institute in Ulanga District, south-eastern Tanzania in 2012, 2013, and 2015

Ward	Mosquito species	2012 *n* (%)	2013 *n* (%)	2015 *n* (%)	Total
Minepa Ward	Total mosquitoes collected	57,393	23,448	39,359	120,200
	*An. arabiensis*, females^a^	15,305 (26.6)	9224 (39.3)	10,950 (27.8)	35,479
	*An. funestus* group, females	7713 (13.4)	1582 (6.7)	3097 (7.9)	12,392
	*Cx. pipiens* complex, males	6469 (11.2)	2062 (8.7)	4160 (10.5)	12,691
	*Cx. pipiens* complex, females^b^	27,906 (48.6)	10,580 (45.1)	21,152 (53.7)	59,638
Mavimba ward	Total mosquitoes collected	44,378	14,673	23,540	82,591
	*An. arabiensis*, females^a^	4292 (9.6)	3158 (21.5)	2101 (8.9)	9551
	*An. funestus* group, females	2460 (5.5)	894 (6.0)	793 (3.4)	4147
	*Cx. pipiens* complex, males	8608 (19.3)	1418 (9.6)	3034 (12.8)	13,060
	*Cx. pipiens* complex, females^b^	29,018 (65.4)	9203 (62.7)	17,612 (74.8)	55,833
Kivukoni ward	Total mosquitoes collected	98,902	34,374	51,251	184,527
	*An. arabiensis*, females^a^	9572 (9.6)	4416 (12.8)	7070 (13.7)	21,058
	*An. funestus* group, females	3327 (3.3)	663 (1.9)	860 (1.6)	4850
	*Cx. pipiens* complex, males	18,905 (19.1)	7546 (21.9)	11,155 (21.7)	37,606
	*Cx. pipiens* complex, females^b^	67,098 (67.8)	21,749 (63.2)	32,166 (62.7)	121,013

^a^Sub-samples of *An. gambiae* complex mosquitoes collected in this area during this period have consistently been 100% *An. arabiensi*s

^b^A sub-sample of 1053 *Culex* mosquitoes were subjected to further morphological examination and identified as *Cx. pipiens* complex

## Discussion

Until this study, the insecticide susceptibility status of non-malaria vectors such as *Culex* spp. was widely unknown in Tanzania, despite the abundance of these mosquito species. The present study investigated the susceptibility of *Cx. pipiens* complex, to insecticides approved by the WHO for vector control. Standard WHO insecticide susceptibility bioassays were conducted separately for female and male *Culex* mosquitoes collected in different seasons and different wards.

Generally, *Culex* mosquitoes were found resistant to a wide range of pyrethroids, lambda-cyhalothrin and deltamethrin, DDT and the carbamate, bendiocarb. However, these species showed susceptibility to organophosphates, such as pirimiphos-methyl and malathion, except for populations from the Mavimba ward, which were resistant to these insecticide classes.

While resistance was widespread across the study sites, lowest mortalities were observed against bendiocarb in Minepa (mortalities of 29% in female and 58% in male *Culex* mosquitoes) and against deltamethrin in Lupiro (8% mortality in female *Culex*). Previous studies have reported that resistance in male *Cx. pipiens* and *An. gambiae* could potentially affect mating competitiveness in nature [50–52]. Additionally, information on insecticide susceptibility of male mosquitoes could be useful when designing interventions primarily against males, e.g. sterile insect technique (SIT) [53, 54], spraying of male swarms with insecticides [34], and use of attractive toxic sugar baits [55, 56].

In this part of south-eastern Tanzania, LLINs impregnated with permethrin remain the primary vector and diseases control interventions [35]. This study confirmed phenotypic resistance to this and also other public health insecticides that are currently not used in the study area. Our results suggest alternative sources of insecticide resistance selective pressure, most likely from agricultural pesticides [57–60]. Indeed, direct observation in the communities revealed an array of chemical classes widely sold and used for crop protection (Matowo et al., unpublished data). Therefore, for effective vector control, an integrated approach with agricultural pest control programmes in the allocation of insecticides is recommended. With reference to *Culex* spp., which also cause considerable biting nuisance in these communities where most people are small-holder farmers [37], the need for integrated pest and vector management and coordination among the public health and agriculture sectors is particularly important.

The significant differences in phenotypic resistance between the neighbouring wards, as revealed in this study, has also been reported for the malaria vector *An. arabiensis* [29], and clearly suggest that selection of insecticide resistance is happening at fine spatial scales and over time, e.g. between small administrative wards, other than variations previously reported between districts and regions [28, 61, 62]. The spatial and seasonal differences in insecticide resistance in *Cx. pipiens* complex species could be influenced by the presence/use of various insecticides at a particular geographical area and time, even though this study did not directly assess the link between the two. These variations signify an important challenge for vector and disease control programmes when choosing insecticides for particular time periods and locations. For example, susceptibility was generally higher in the wet season than in the dry season. Thus, the possibility that insecticide-based interventions aimed at the wet season may have greater entomological and epidemiological impact on mosquito densities and disease transmission than those in the dry season, should be investigated. These fine-scale spatial and temporal variations are increasingly being reported across multiple sites. In one study in a single area in Mexico, both resistance phenotypes and genotypes were markedly varied at a fine spatial scale and time, in *Aedes aegypti* populations against chorpyrifos-ethyl and deltamethrin, driven by fine-scale pressure from the household insecticides use [63]. Similarly, Grossman et al. [64] recently reported spatial and seasonal heterogeneity in the frequency of *kdr* haplotypes in *Ae. aegypti* from Mexico, likely to be influenced by differences in the usage of insecticides in space and time. In Uganda, there were monthly variations in *kdr* allele frequency in *Plasmodium-*infected *An. gambiae* (*s.s.*) and the resistance was significantly higher in the dry compared to the wet season which is likely to be caused by seasonal changes in insecticide pressure [65]. A recent report by Jones et al. [22] on insecticide resistance in *Cx. quinquefasciatus* from Zanzibar showed variability of resistance levels between nearby study sites, though the results were incomparable due to differences of *Culex* species at these sites. Niang et al. [66] also reported spatial variations of the L1014F *kdr* allele found to dominate in *An. arabiensis* compared to *An. coluzzii* and *An. gambiae* sampled from 20 different study sites in the south-eastern part of Senegal.

Mechanisms for resistance appear to be mixed. The partial suppression of pyrethroid resistance by synergist PBO and TPP exposures suggests that both P450 monooxygenases and esterases might be contributing to the pyrethroid resistance phenotypes observed in mosquito populations sampled from both Minepa and Mavimba wards. However, esterases seemed not to be involved in lambda-cyhalothrin resistance in mosquitoes from the Minepa ward, as only minimal change in mortality was observed upon pre-exposure to the synergist. In addition, DDT resistance was significantly restored after being exposed to DEM, suggesting a role for GSTs in DDT resistance in the Mavimba ward. However, DDT was not affected by DEM in Minepa samples, suggesting no role for metabolic resistance mechanisms here. Besides metabolic resistance, other resistance mechanisms, such as *kdr* mutation, could play a role and further research is required to identify the mechanisms of resistance. These observations are consistent with previous studies on incomplete suppressions of pyrethroids and DDT resistances due to pre-exposure to synergists [22, 30, 31]. Nonetheless, the multiplicity of resistance mechanisms in these mosquito populations is a major concern and should be considered by control programmes.

In line with the WHO Global Vector Control Response strategy [18], it is important to integrate control of different arthropod vectors. In this area, where malaria is certainly the most important mosquito-borne disease, 79% of biting risk indoors was associated with *Cx. pipiens* mosquitoes. Despite long-term use of the permethrin-based Olyset® nets, which are regularly distributed *via* the national government’s mass distribution campaigns [35], *Culex* mosquitoes were fortunately found susceptible to permethrin, except in the Lupiro ward in the wet season (Table 3). Nonetheless, as resistance continues to spread, additional approaches, such as improved housing, larval source management, and indoor residual spraying (IRS) with non-pyrethroids and non-carbamates, may be considered as alternatives against both *Culex* and malaria vectors.

The most abundant *Culex* species in tropical and subtropical countries, including East Africa, belong to the *Cx. pipiens* complex, which contains *Cx. quinquefasciatus*, *Cx. pipiens pipiens*, *Cx. pipiens torrentium* and *Cx. pipiens molestus* [3, 4]. From our findings, 94% of *Culex* belonged to the *Cx. pipiens* complex, of which 81% were verified by PCR as *Cx. quinquefasciatus*, 2% as *Cx. pipiens pipiens* and 3% as hybrids of *Cx. pipiens pipiens* and *Cx. quinquefasciatus.* A small proportion (14%) of samples were non-amplified, suggesting other *Culex* species for which there were no primers to distinguish. The presence of hybrids of *Cx. pipiens pipiens* and *Cx. quinquefasciatus* suggests that these species cross-mate in the wild. *Cx. quinquefasciatus* was previously documented through morphological identification as the dominant *Culex* species in the Kilombero Valley, where it occurred alongside a few *Cx. theileri* and *Cx. univittatus* [1]. However, none of these species were confirmed by PCR [1].

An important limitation of this study is that we analysed only a relatively small number of *Culex* to identify other possible *Culex* sibling species using PCR techniques due to the lack of appropriate primers. It should also be noted that synergists findings presented here stem from only female *Culex* specimens sampled in the dry season in Minepa and Mavimba wards as synergist papers are not easily accessible and not produced in bulk.

## Conclusions

*Culex* *pipiens* complex, which mostly consists of *Cx. quinquefasciatus*, are the most abundant *Culex* species in the study area, and contribute to more than 79% of all biting risk experienced in houses. The species are resistant to carbamates and pyrethroids commonly used in public health inside houses and also to DDT. The organophosphate, pirimiphos-methyl, which is also available for IRS however remains effective. This study has also demonstrated that insecticide resistance phenotypes and the underlying mechanisms varies considerably at fine geographical scales, suggesting some modifications to current insecticide resistance monitoring plans. Monooxygenases and esterases partly underlie the resistance phenotypes against pyrethroids, while GSTs play an important role in DDT resistance. Further investigations are required to identify more drivers and other mechanisms of resistance in *Culex* species across the wards. Overall, the extent of resistance reported in the current study indicates that additional approaches, such as improved housing, community-based larval source management, and IRS with non-pyrethroids and non-carbamates, should be considered as complementary vector control strategies. Lastly, resistances against insecticides not currently used for vector control in the villages, suggests possible linkages to agricultural pesticides use. Hence, multi-sectorial approaches should be encouraged to improve management of insecticide resistance.

## Data Availability

The datasets used and/or analysed during the current study are available from the corresponding author upon reasonable request.
